# Plasmid-Based CRISPR-Cas9 Gene Editing in Multiple *Candida* Species

**DOI:** 10.1128/mSphere.00125-19

**Published:** 2019-03-13

**Authors:** Lisa Lombardi, João Oliveira-Pacheco, Geraldine Butler

**Affiliations:** a School of Biomolecular and Biomedical Science, Conway Institute, University College Dublin, Belfield, Dublin, Ireland; Carnegie Mellon University

**Keywords:** CRISPR, *Candida*, genome editing

## Abstract

Many *Candida* species that cause infection have diploid genomes and do not undergo classical meiosis. The application of clustered regularly interspaced short palindromic repeat-Cas9 (CRISPR-Cas9) gene editing systems has therefore greatly facilitated the generation of gene disruptions and the introduction of specific polymorphisms. However, CRISPR methods are not yet available for all *Candida* species. We describe here an adaption of a previously developed CRISPR system in Candida parapsilosis that uses an autonomously replicating plasmid. Guide RNAs can be introduced in a single cloning step and are released by cleavage between a tRNA and a ribozyme. The plasmid also contains *CAS9* and a selectable nourseothricin *SAT1* marker. It can be used for markerless editing in C. parapsilosis, C. orthopsilosis, and C. metapsilosis. We also show that CRISPR can easily be used to introduce molecular barcodes and to reintroduce wild-type sequences into edited strains. Heterozygous mutations can be generated, either by careful selection of the distance between the polymorphism and the Cas9 cut site or by providing two different repair templates at the same time. In addition, we have constructed a different autonomously replicating plasmid for CRISPR-Cas9 editing in Candida tropicalis. We show that editing can easily be carried out in multiple C. tropicalis isolates. Nonhomologous end joining (NHEJ) repair occurs at a high level in C. metapsilosis and C. tropicalis.

**IMPORTANCE** *Candida* species are a major cause of infection worldwide. The species associated with infection vary with geographical location and with patient population. Infection with Candida tropicalis is particularly common in South America and Asia, and Candida parapsilosis infections are more common in the very young. Molecular methods for manipulating the genomes of these species are still lacking. We describe a simple and efficient CRISPR-based gene editing system that can be applied in the C. parapsilosis species group, including the sister species Candida orthopsilosis and Candida metapsilosis. We have also constructed a separate system for gene editing in C. tropicalis.

## Abstract (precis)

*Candida* species are a major cause of infection worldwide. The species associated with infection vary with geographical location and with patient population. Infection with Candida tropicalis is particularly common in South America and Asia, and Candida parapsilosis infections are more common in the very young. Molecular methods for manipulating the genomes of these species are still lacking. We describe a simple and efficient CRISPR-based gene editing system that can be applied in the C. parapsilosis species group, including the sister species Candida orthopsilosis and Candida metapsilosis. We have also constructed a separate system for gene editing in C. tropicalis.

## INTRODUCTION

Opportunistic yeast pathogens, including *Candida* species, cause a wide variety of infections, ranging from superficial to systemic, which can often be fatal (1). Infection of premature neonates, the elderly, and immunocompromised patients is particularly common (2). Extensive use of catheters, broad-spectrum antibiotics, and abdominal surgery also favors the spread of pathogenic yeasts from their normal commensal niches (2).

More than 30 *Candida* species are known to cause disease (3, 4). Although C. albicans is the most common cause of candidiasis, the emergence of non-*albicans Candida* species such as Candida dubliniensis, Candida glabrata, Candida krusei, Candida parapsilosis, and Candida tropicalis has increased over the past decades (5, 6), and Candida auris has recently been reported as an emerging multidrug-resistant species (7, 8). Many of the well characterized *Candida* species, including C. albicans, C. parapsilosis, and C. tropicalis, belong to the CUG-Ser1 clade, in which the CUG codon is translated as serine rather than leucine (9, 10). C. tropicalis is particularly prevalent in South America and Asia (4). Infections by members of the C. parapsilosis group, which includes Candida orthopsilosis and Candida metapsilosis, are most common in the very young and the very old (11).

C. albicans and C. tropicalis and the C. parapsilosis species complex have diploid genomes and do not undergo meiosis, which makes generating deletion strains a difficult process. Each allele needs to be targeted independently. Several gene deletion methods based on homologous recombination were developed for C. albicans, including sequential replacement of alleles with a recyclable marker (12, 13) or with different markers in an auxotrophic background (14). Some systems were adapted for use in C. parapsilosis (15–17) and C. tropicalis (18–21). Recently, counterselection against the *mazF* gene of Escherichia coli was used to make markerless disruptions in C. tropicalis (22). The recent discovery of haploid forms of C. albicans has enabled disruption strategies in this species that are not yet applicable in the others (23–25).

The advent of clustered regularly interspaced short palindromic repeat (CRISPR)-based gene editing tools has revolutionized studies in many *Candida* species, including C. albicans, C. glabrata, C. lusitaniae, and C. auris (7, 26–37). Various approaches have been used, including integrating *CAS9* in the genome (see, e.g., reference 26) and transient expression of *CAS9* (33) and providing Cas9 as part of an RNA-protein complex (36, 37). Some systems require cloning (see, e.g., reference 27), and some can be constructed using only PCR (see, e.g., references 32 and 33). Some introduce markers at the target site that can be subsequently removed (see, e.g., reference 32). We recently described a CRISPR-Cas9 system on a replicating plasmid that can be used for markerless gene editing in C. parapsilosis (29). The *CAS9*-containing plasmid is quickly lost in the absence of selection. The system has also been applied in C. orthopsilosis (38). Although the plasmid-based gene editing method is efficient, two cloning steps were required, which means that it was not readily applicable to large-scale efforts. Here, we adapted the plasmid system so that the guide RNA (gRNA) was able to be introduced in a single cloning step. The system can also be used for gene editing in C. orthopsilosis and C. metapsilosis. Furthermore, we made a new plasmid for CRISPR editing in C. tropicalis. We show that with careful guide design, CRISPR can be used to generate heterozygous variants.

## RESULTS AND DISCUSSION

### Modification of plasmid-based CRISPR-Cas9 gene editing in C. parapsilosis.

**FIG 1:**
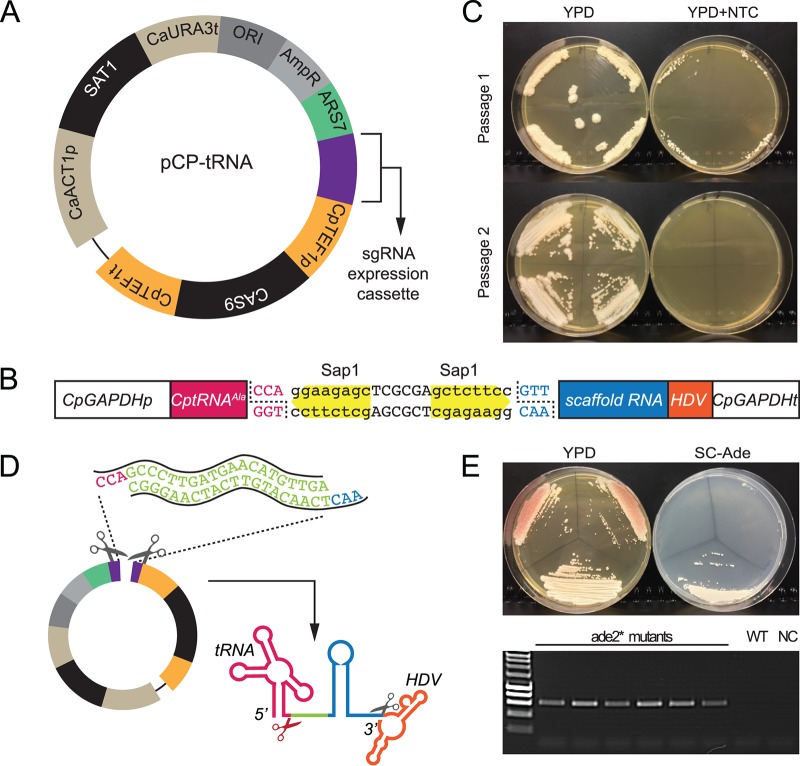
The pCP-tRNA plasmid system for gene editing in C. parapsilosis. (A) The plasmid shares the main features of the pRIBO system (29), namely, the *SAT1* gene (nourseothricin resistance), autonomously replicating sequence 7 (*ARS7*) from C. parapsilosis, and the *CAS9* gene expressed from the C. parapsilosis *TEF1* promoter. (B) The pRIBO and pCP-tRNA systems differ in the cassette used to express the sgRNA. In pCP-tRNA, the RNA pol II GAPDH promoter is followed by the tRNA^Ala^ sequence (in pink), two SapI restriction sites (in yellow), the scaffold RNA (in blue), and the hepatitis delta virus (HDV) sequence (in orange). (C) Like pRIBO, pCP-tRNA is easily lost. Transformed cells were patched to YPD plates without nourseothricin (NTC) for 48 h and were then streaked on YPD and YPD plus NTC. Colonies from YPD were repatched after 48 h. All transformants lost NTC resistance after just two passages. (D) The target guide (in green, representing ADE2-B in panel E) was generated by annealing two 20-bp oligonucleotides carrying overhang ends (in pink and blue) and cloned into SapI-digested pCP-tRNA. The guide RNA is released by cleavage after the tRNA^Ala^ and before the HDV ribozyme. (E) Editing of *ADE2* using the pCP-tRNA system. Transformation of C. parapsilosis CLIB214 with pCP–tRNA–ADE2-B and a repair template (RT-B [29]) resulted in the introduction of two stop codons that disrupted the gene function, producing pink colonies that failed to grow in the absence of adenine (SC-ade). A white Ade-positive (Ade^+^) wild-type colony is shown as a control. The transformants were screened by PCR using the mutADE2B-F primer derived for the edited site and the downstream ADE2_REV primer, which generates a product only when the mutation is present as described in reference 29. WT (wild type), CLIB214 strain; NC, no DNA.

We first described a plasmid-based CRISPR-Cas9 editing system in C. parapsilosis in 2017 (29). The guide RNA is introduced between two ribozymes (Hammerhead and hepatitis delta virus [HDV] ribozymes) in a two-step cloning process and is released by self-cleavage of the ribozymes. Changing the guide RNA to target a new gene also requires changing bases in the Hammerhead ribozyme. Here, we replaced the Hammerhead ribozyme with a tRNA^Ala^ sequence from C. parapsilosis using a synthetic construct, based on systems described by Ng and Dean (39) (pCP-tRNA) (Fig. 1A). The gRNA is now introduced in a single step by designing two 20-base oligonucleotides with overhanging ends compatible with two SapI sites (Fig. 1B). The mature single guide RNA (sgRNA) molecule is released by cleaving after the tRNA^Ala^ by endogenous yeast RNase Z endonuclease and self-splicing before the HDV ribozyme (Fig. 1D). The plasmid expresses *CAS9* and contains a selectable marker (nourseothricin resistance; Fig. 1A). Plasmid loss is induced after only two passages in the absence of selection (Fig. 1C). Gene editing is carried out in a single transformation step, by introducing the plasmid together with a repair template (RT). Repair templates (described in reference 29) are generated by overlapping PCR and include homology arms that are 50 and 34 bp upstream and downstream of the cut site, respectively. Fig. 1E shows editing of C. parapsilosis *ADE2* (*CpADE2*) using pCP-tRNA by the introduction of two stop codons, using sgADE2-B as previously described by Lombardi et al. (29). Disruptions in *ADE2* are easily detected because they accumulate a red/pink pigment on yeast extract-peptone-dextrose (YPD) media, due to a defect in adenine biosynthesis. Transformants were screened by PCR using one primer specific to the edited site followed by sequencing (see Table S2 at https://doi.org/10.6084/m9.figshare.7776842). The efficiency of editing is comparable to that seen with the original system (approximately 80%).

**FIG 2:**
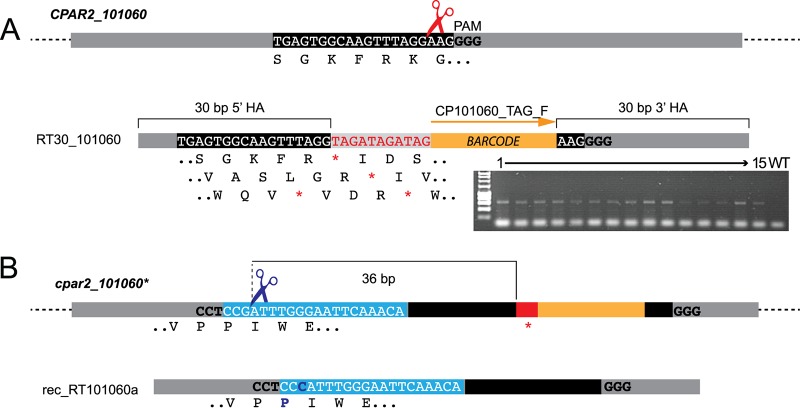
Editing and reconstitution of *CPAR2_101060*. (A) The plasmid pCP-tRNA-CP101060 was generated to target *CPAR2_101060*. The guide sequence recognized by Cas9 is boxed in black, and the PAM is shown in bold. The Cas9 cut site is indicated by red scissors. C. parapsilosis CLIB214 cells were transformed with pCP-tRNA-CP101060 and a repair template (RT30_101060) generated by overlapping PCR using RT30_101060_TOP and RT30_101060_BOT oligonucleotides. The repair template contains two 30-bp homology arms (HA) that flank an 11-bp sequence containing coding stop codons in all three possible reading frames (in red, with all reading frames indicated below the sequence) and a 20-bp unique barcode (in orange). The gel shows results of screening of 15 transformants by PCR using primer CP101060_TAG_F, which anneals to the barcode, together with the CP101060_WT_R downstream primer. Sequencing confirmed that stop codons were introduced into both alleles of *CPAR2_101060*. (B) To replace the cpar2_101060* edited alleles with wild-type sequences, a PAM site (bold) upstream from the edited site (red) was selected. The guide RNA is boxed in light blue. Transformation with pCP-rec-tRNA-Cp101060a containing this guide resulted in Cas9 cleavage 36 bp upstream from the mutated region (indicated by blue scissors). The repair template (rec-RT101060a) generated by overlapping PCR with primers rec-RT-101060aTOP and rec-RT-101060aBOT was designed to replace the edited site and barcode with wild-type sequences. It also included a single G1420C synonymous SNP so that the reconstituted and wild-type alleles could be distinguished. The wild-type sequence was successfully reintroduced in 2/9 transformants tested. The scheme is not drawn to scale.

We next explored the possibility of introducing unique molecular barcodes at the edited site while retaining short repair templates. We targeted the gene *CPAR2_101060* as proof of principle, using a repair template with 30 bp of homology arms flanking 11 bp containing stop codons in all open reading frames and a unique tag (Fig. 2A). PCR screening of 15 transformants showed that the barcode was incorporated into all 15 (Fig. 2A). The edited site was confirmed by sequencing. This approach will be useful in large-scale studies, as it results in the disruption of the gene regardless of the reading frame and can be used to specifically barcode each mutant strain for use in competition studies. We also showed that short (30-bp) regions can be used to drive homologous recombination in C. parapsilosis.

Nguyen et al. (32) showed that CRISPR-Cas9 can be used to reconstitute mutated strains in C. albicans by reintroducing the wild-type sequence at the native locus. In that study (32), the edited sequence included a new protospacer adjacent motif (PAM) site that was targeted with a common gRNA. To avoid adding even more sequences at the edited site, we instead selected a naturally occurring PAM site 36 bp upstream from the edited region (Fig. 2B). A synonymous single nucleotide polymorphism (SNP) (G1420C) was introduced into the repair template so that we could distinguish the reconstituted strain from the original wild-type strain (Fig. 2B). Among 9 sequenced transformants, 2 contained the reconstituted wild-type sequence, removing the stop codon and the barcode from both alleles and introducing the synonymous SNP (reconstituted allele *REC101060*; see Table S1 at https://doi.org/10.6084/m9.figshare.7776833). The remaining 7 all contained the synonymous SNP, but they also retained the edited region and barcode, possibly due to the lower efficiency of homologous recombination corresponding to increasing distance from the Cas9 cut site, as discussed below (40).

### CRISPR-Cas9 gene editing in C. metapsilosis.

**FIG 3:**
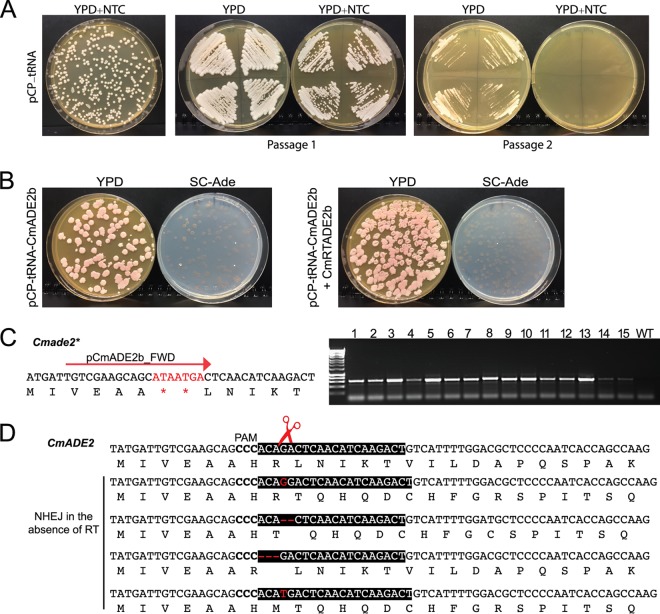
Editing of *ADE2* in C. metapsilosis. (A) Plasmid pCP-tRNA propagates in C. metapsilosis SZMC8093, and it is lost after two passages on YPD in the absence of nourseothricin (NTC, 200 μg/ml). (B) C. metapsilosis was transformed with plasmid pCP-tRNA-CmADE2b, targeting *CmADE2* either without (left side) or with (right side) a repair template (CmRTADE2b, generated by overlapping PCR with primers CmRTADE2b_TOP/CmRTADE2b_BOT) designed to introduce two stop codons. The transformants were replica plated on YPD and Sc-Ade. Almost all colonies were pink and were unable to grow in the absence of adenine. (C) In the presence of the repair template, most transformants contained the inserted stop codons, identified by PCR using primers pCmADE2b_FWD and CmADE2_REV. Results of colony PCR of 15 representative colonies are shown; more are shown in Fig. S1 at https://doi.org/10.6084/m9.figshare.7776761. The wild-type (WT) strain was included as a control. (D) Many pink transformants were obtained even in the absence of the repair template. Sequencing of the region surrounding the Cas9 cut site revealed a variety of repair events, including insertions and deletions (indicated in red), resulting in either frameshift or deletion of His23. These presumably resulted from NHEJ.

Zoppo et al. (38) showed that the original plasmid designed for CRISPR editing in C. parapsilosis can be used to edit genes in C. orthopsilosis, and the tRNA-based plasmid is also effective in this species (F. Morio, L. Lombardi, U. Binder, C. Logé, E. Robert, D. Graessle, M. Bodin, C. Lass-Flörl, G. Butler, P. Le Pape, unpublished data). We therefore tested the pCP-tRNA system in C. metapsilosis, the third member of the C. parapsilosis species group, by targeting *CmADE2*. Unlike C. parapsilosis, where heterozygosity levels are low, all C. metapsilosis isolates characterized to date descended from hybridization between two parental strains that differed by about 5% at the sequence level (41). The pCP-tRNA plasmid is easily introduced into C. metapsilosis SZMC8093, where it propagates without integration. Similarly to C. parapsilosis, nourseothricin resistance is quickly lost in the absence of selection (Fig. 3A). Cells transformed with a plasmid targeting *CmADE2* and a repair template carrying 35 and 48 bp of upstream and downstream homology regions designed to introduce two stop codons were edited with 100% efficiency. All transformants were pink when replica plated on YPD and failed to grow on synthetic media in the absence of adenine (Fig. 3B, right plate). PCR analysis of 95 transformants (see Fig. S1 at https://doi.org/10.6084/m9.figshare.7776761) using a allele specific primer showed that 88 had the expected mutation; that result was confirmed by sequencing 6 representative transformants. Repair by homologous recombination therefore occurs at a high rate in C. metapsilosis when a suitable repair template is provided. Interestingly, in the absence of the repair template, most colonies were also pink and adenine auxotrophs. Sequencing of 12 transformants showed that a variety of nonhomologous end joining (NHEJ)-like repair events had occurred, including deletion of either 2 or 3 nucleotides and insertion of 1 nucleotide, resulting in either a frameshift or the deletion of one amino acid (Fig. 3D). This suggests that repair via NHEJ is common in C. metapsilosis (Fig. 3D). All the NHEJ repair events were identical in the two alleles, indicating that NHEJ may have occurred at one allele, followed by a homology-directed repair (HDR) event at the second allele.

### Designing a plasmid for gene editing in C. tropicalis.

**FIG 4:**
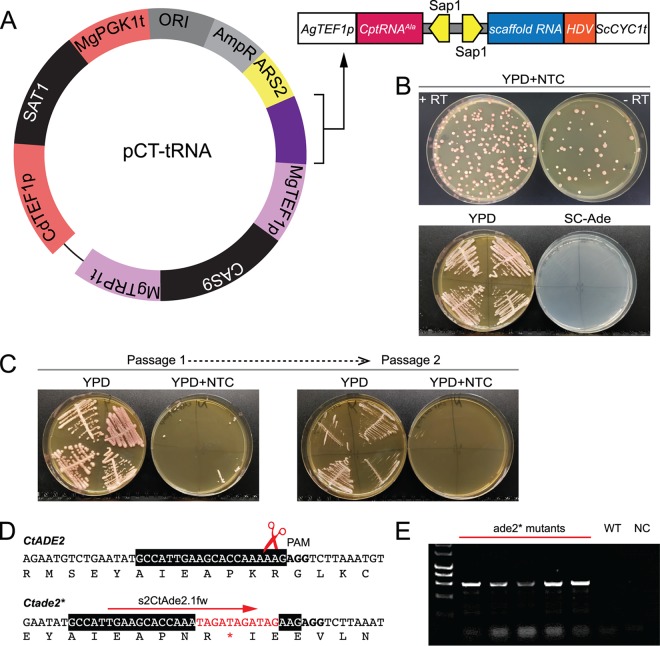
The pCT-tRNA plasmid system for gene editing in C. tropicalis. (A) The plasmid shares the main features of the pCP-tRNA system, except that different regulatory elements and a different autonomously replicating sequence are used. The *SAT1* gene (nourseothricin resistance) is flanked by a *CdTEF1* promoter and a *MgPGK1* terminator, and the *CAS9* gene is expressed from the M. guilliermondii *TEF1* promoter. Autonomously replicating sequence 2 (*ARS2*) was derived from C. albicans (43, 44). The cassette for the expression of the sgRNA is highlighted in purple on the plasmid map and is represented in more detail in the scheme on the right side. The color coding is the same as in Fig. 1. The only differences from the cassette in Fig. 1B are the promoter (*AgTEF1p*) and the terminator (*ScCYC1t*). (B) Editing of *CtADE2* using the pCT-tRNA system. The guide gCtADE2.1 was generated by annealing CtAde2.1_gTOP and CtAde2.1_gBOT oligonucleotides (see Table S2 at https://doi.org/10.6084/m9.figshare.7776842) and was cloned into SapI-digested plasmid pCT-tRNA to generate pCT-tRNA-CtADE2.1. Transformation of C. tropicalis Ct46 with pCT-tRNA-CtADE2.1 and the R60-CtADE2-b repair template resulted in the introduction of a stop codon that disrupted the gene function, producing pink auxotrophs. Pink colonies were also observed when cells were transformed with pCT-tRNA-CtADE2.1 without any repair template, presumably due to NHEJ-like repair events (see also Fig. S3 at https://doi.org/10.6084/m9.figshare.7776824). (C) pCT-tRNA-CtADE2.1 is easily lost. Representative pink colonies were patched to YPD plates without nourseothricin (NTC) for 48 h and were then streaked on YPD and YPD plus NTC. Colonies from YPD were repatched after 48 h. All transformants lost NTC resistance after just two passages. (D) The transformants were screened by PCR using the s2CtAde2.1fw primer derived from the edited site and the downstream pCtADE2.1_REV primer, which generates a product only when the mutation is present. (E) Result of PCR screening of 5 representative transformants. WT, Ct46 strain; NC, no DNA.

Some advantages of using a replicating plasmid-based gene editing system are that any gene can be edited in any isolate in a markerless way. Currently, there are few methods available for editing in *Candida* species other than C. albicans and the C. parapsilosis species group. We found that the pCP-tRNA plasmid failed to generate nourseothricin-resistant transformants of C. tropicalis. We therefore adapted constructs designed by Defosse et al. (42), who previously identified suitable promoters and terminators for use in C. tropicalis. We first replaced the *GFP* gene in pAYCU268 from Defosse et al. (42) with *CAS9* from Vyas et al. (27), placing *CAS9* under the control of the *TEF1* promoter from Meyerozyma guilliermondii. pAYCU268 expresses *SAT1* from the C. dubliniensis *TEF1* promoter and is designed to integrate randomly into the C. tropicalis genome. We identified an autonomously replicating sequence (CaARS2) which is reported to promote replication in C. tropicalis (43, 44) and introduced it into the pAYCU268 backbone. Finally, a tRNA cassette similar to that in pCP-tRNA was synthesized and inserted into the plasmid, generating pCT-tRNA (Fig. 4A). Guide RNAs were then able to be cloned between the tRNA and a ribozyme and were expressed from an Ashbya gossypii *TEF1* promoter, followed by the Saccharomyces cerevisiae *CYC1* terminator. An important feature of this plasmid is that, apart from CaARS2, all the DNA parts can be changed thanks to the presence of specific restriction sites.

The pCT-tRNA plasmid containing a guide RNA targeted against *CtADE2* was used to transform 5 different isolates of C. tropicalis, together with a repair template designed to introduce one stop codon in each frame (Fig. 4D; see also Fig. S3 at https://doi.org/10.6084/m9.figshare.7776824). The repair template has 60-bp homology arms flanking the cleavage site and was generated by overlapping PCR. Pink auxotrophs were observed following transformation of each isolate, in the presence or absence of repair template, with efficiency ranging from 88% to 100% (Fig. 4B) (Table 1). PCR screening and subsequent sequencing analysis of representative transformants obtained using the repair template confirmed the presence of the inserted stop codons (Fig. 4D and E). In the absence of the repair template, pink adenine auxotrophs resulted from the deletion of one base near the Cas9 cut site (Fig. 4B; see also Fig. S3A at https://doi.org/10.6084/m9.figshare.7776824) (Table 1). The same deletion was found in both alleles. NHEJ-like repair events, possibly followed by HDR, therefore occur at a high frequency in C. tropicalis. Just like pCP-tRNA, the pCT-tRNA plasmid is rapidly lost from transformants grown in the absence of selection (Fig. 4), thus minimizing the risk of Cas9 cutting at off-target sites.

**TABLE 1:** Efficiency of CRISPR-based editing with the pCP/CT-tRNA systems in *C. parapsilosis*, *C. metapsilosis*, and *C. tropicalis*

Strain	Target gene	% edited transformants^*a*^			
		−RT		+RT	
		Expt 1	Expt 2	Expt 1	Expt 2
C. parapsilosis					
CLIB214	*CpADE2*	No colonies	No colonies	75	80
CLIB214	*CPAR2_101060*	No colonies	ND	100	ND

C. metapsilosis					
SZMC8093	*CmADE2*	100	ND	100	ND

C. tropicalis					
DSM4959 (ct44)	*CtADE2*	100	94	63	50
CAS08-0047 (ct45)	*CtADE2*	92	100	100	100
CAS08-0093 (ct46)	*CtADE2*	97	94	85	71
CAS08-0098 (ct47)	*CtADE2*	92	91	77	88
CAS08-0102 (ct48)	*CtADE2*	88	95	95	91

a Data represent mutation efficiencies determined in one or two experiments. The mutation efficiency of *ADE2* was calculated based on color and inability to grow in the absence of adenine. In the case of *CPAR2_101060*, the mutation efficiency was calculated based on PCR screening. RT, repair template; ND, not determined.

### Using CRISPR to introduce heterozygous mutations.

One of the problems with CRISPR editing of diploid genomes is that it tends to be an all-or-nothing affair—either both alleles are edited or neither allele is. This can be a problem with essential genes, where editing both alleles would be lethal. Vyas et al. (26) approached this problem by generating temperature-sensitive alleles in C. albicans. However, Paquet and colleagues (40) recently described how the design of the repair template can be used to introduce mutations into just one allele in the genome of human cells. They showed that there is an inverse relationship between the efficiency of incorporation of a desired SNP and its distance from the Cas9-induced double-strand break. This can be exploited to push the editing system toward the introduction of mutations at one allele only. Alternatively, cells transformed with a mixture of two different repair templates can incorporate a different template at each allele (40). We tested if the same approaches are effective in C. parapsilosis by targeting the (nonessential) *CPAR2_101060* gene.

**FIG 5:**
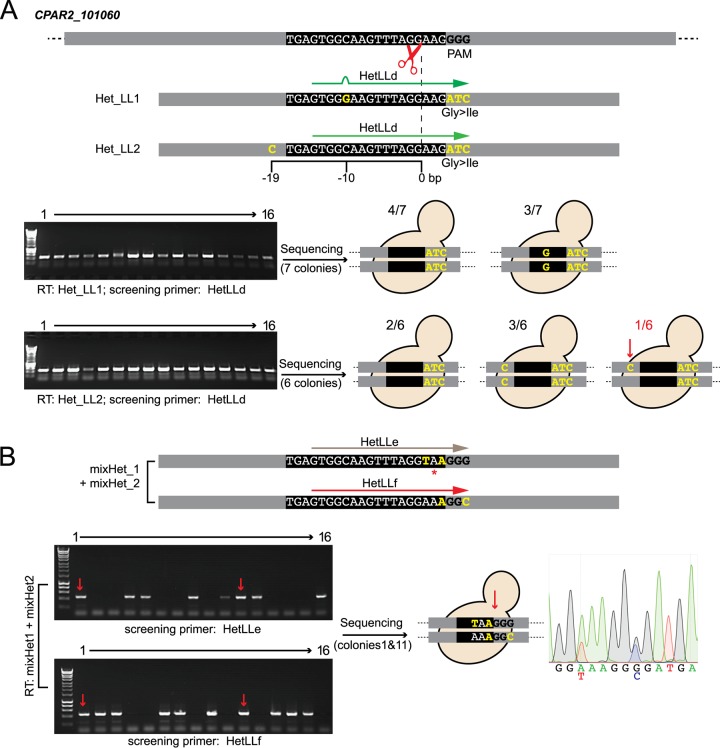
Generation of heterozygous mutations with the CRISPR-Cas9 system. (A) Varying the distance between the Cas9 cut site and introduced DNA change. (B) Using a mixture of repair templates. (A) C. parapsilosis CLIB214 cells were transformed with pCP-tRNA-CP101060 and either the Het_LL1 repair template or the Het_LL2 repair template. Both Het_LL1 and Het_LL2 carry three nucleotide changes that introduce a codon change and disrupt the PAM (GGG to ATC, resulting in a Gly-to-Ile amino acid change) (yellow). The two templates also carry an additional SNP which introduces a silent mutation at either 10 bp (C>G) or 19 bp (T>C) upstream from the Cas9 cut site. Sixteen transformants obtained with Het_LL1 and Het_LL16 transformed with Het_LL2 yielded a PCR product using primer HetLLd, which anneals at the ATC codon change, and the CP101060_WT_R downstream primer. HetLLd does not anneal perfectly to the sequence introduced by Het_LL1, because of the C>G SNP. The cartoons show the sequencing results. With repair template Het_LL1, all 7 transformants contained the amino acid change at both alleles; in 3 transformants, G was introduced at both alleles 10 bp upstream of the cut site, and four retained the wild-type C base. With repair template Het_LL2, all 6 transformants again contained the amino acid change at both alleles; 5 transformants contained either the wild-type base or the mutated base at the additional site, but one was heterozygous for a T>C SNP 19 bp from the cut site. The combination of alleles in this strain is referred to as *CP101060-ATC*/*CP101060-ATC-SNP.* (B) C. parapsilosis CLIB214 was transformed with pCP-tRNA-CP101060 and a mixture of two repair templates, mixHet_1 and mixHet_2. MixHet_2 is designed to introduce two silent mutations that do not change the coding sequence but that do disrupt the PAM site, preventing Cas9 from cutting again at the edited site. mixHet_1 also contains two mutations which change the protospacer preventing the gRNA binding and which introduce a stop codon. Among the 16 transformants, 2 (indicated by the red arrows) generated PCR products when amplified with primers annealing to either of the targeted editing sites (HetLLe or HetLLf) and the CP101060_WT_R downstream primer. Sequencing of these colonies confirmed that different repair templates had been incorporated at each allele. The chromatogram shows one example. Genotype: *CP101060-Stop*/*CP101060-SNP*.

Both strategies were used. In strategy 1, C. parapsilosis CLIB214 was transformed with a plasmid targeting *CPAR2_101060* and with one of two repair templates. Both repair templates carry three base substitutions (GGG>ATC) that disrupt the PAM and that introduce a Gly154Ile amino acid change. In addition, one repair template contains one synonymous SNP (C>G) 10 bp upstream from the Cas9 cut site, and the second contains a different synonymous SNP (T>C) 19 bp upstream from the cut site (Fig. 5A; RTs Het_LL1 and Het_LL2, respectively). All the transformants obtained with either repair template that were tested by PCR contained the ATC amino acid change that also disrupts the PAM (Fig. 5A). Seven transformants obtained with the first repair template (Het_LL1) were all homozygous (either C or G) at the position 10 bp upstream from the cut site (Fig. 5A, upper panel). Using the second repair template (Het_LL2), 2 of 6 transformants tested were homozygous for the wild-type nucleotide, 3 incorporated C at both alleles, and 1 was heterozygous, with T at one allele and C at the second (Fig. 5A, lower panel). Increasing the mutation-to-cut site distance can therefore increase the chances of obtaining heterozygous substitutions in C. parapsilosis. It is probably necessary to disrupt the PAM site or the protospacer when using this strategy to prevent repeated recutting by Cas9.

In strategy 2, two repair templates (mixHet_1 and mixHet_2; Fig. 5B) were supplied simultaneously. Both were designed to introduce synonymous SNPs that disrupt either the PAM or the protospacer. One repair template also contained a stop codon immediately upstream of the PAM. Incorporation of one repair template at one allele and the other at the second allele should generate a functional heterozygote, in which one allele has a stop codon and one does not. PCR screening showed that one repair template or other was incorporated into 14 of 16 transformants tested (Fig. 5B). For two colonies, PCR and sequence analysis showed that both repair templates were incorporated, with a stop codon introduced at only one allele (Fig. 5B). The presence of the synonymous SNPs at both alleles indicated that homology-directed repair had occurred at both, with one repair using repair template 1 and the other repair using template 2. Two different strategies can therefore be used in combination with the pCP-tRNA plasmid to introduce heterozygous mutations.

### Conclusion.

The pCP/CT-tRNA systems can be used for CRISPR-Cas9-mediated gene editing in all three members of the C. parapsilosis *sensu lato* complex, and in C. tropicalis. CRISPR editing can also be used to reconstitute wild-type alleles and to generate heterozygous mutations.

## MATERIALS AND METHODS

### Strains and media.

All C. parapsilosis, C. metapsilosis, and C. tropicalis strains used in this study (see Table S1 at https://doi.org/10.6084/m9.figshare.7776833) were grown in YPD medium (1% yeast extract, 2% peptone, 2% dextrose) or on YPD plates (YPD plus 2% agar) at 30°C. Transformants were selected on YPD agar supplemented with 200 μg/ml nourseothricin (Werner Bioagents, Jena, Germany). Auxotrophies were confirmed by growing mutant strains on synthetic complete (SC) dropout media (0.19% yeast nitrogen base without amino acids and ammonium sulfate, 0.5% ammonium sulfate, 2% glucose, 0.075% amino acid dropout mix, 2% agar). All the plasmids used in this study (see Table S3 at https://doi.org/10.6084/m9.figshare.7776845) were propagated in Escherichia coli DH5α cells (NEB, United Kingdom) by growing cells in LB media without NaCl (Formedium) supplemented with 100 μg/ml ampicillin (Sigma).

### Construction of the pCP-tRNA series plasmids.

The synthetic construct GAPDHp-tRNA-SapI-HDV (Eurofins MWG; see Fig. S2 at https://doi.org/10.6084/m9.figshare.7776818) was designed to include the C. parapsilosis glyceraldehyde-3-phosphate dehydrogenase (GAPDH) gene (*GAPDH*) (*CPAR2_808670*) promoter, a C. parapsilosis tRNA^Ala^ sequence, two tandem SapI/BspQI sites for the cloning of the guide, a hepatitis delta virus (HDV) ribozyme, and the *GAPDH* terminator. The cassette was cloned by Gibson assembly (primers are listed in Table S2 at https://doi.org/10.6084/m9.figshare.7776842) into NruI-digested pSAT3 plasmid, which differs from the published pSAT1 plasmid (29) only in that it does not contain any SapI/BspQI sites. The guide RNA was generated by annealing of two 23-bp oligonucleotides carrying appropriate overhanging ends and was cloned into the SapI-digested pCP-tRNA plasmid (see Table S2 at https://doi.org/10.6084/m9.figshare.7776842 and Table S3 at https://doi.org/10.6084/m9.figshare.7776845).

### Construction of pCT-tRNA plasmids.

pAYCU268, from Defosse et al. (42), was used as a starting vector. The green fluorescent protein gene (*GFP*) was replaced with *CAS9* from pV1326 (27) by Gibson assembly using primers jpGA_pAYCU268.fw and GA_pAYCU.CAS9.rv to amplify the backbone and primers jpGA_CAS9.1326.fw and pGA_Cas9.1326.rv to amplify *CAS9*, producing the plasmid GA_pAYCU.CAS9.1326. CaARS2 (43) was amplified using V3_GA.CaARS2.1326.fw and V3_GA.CaARS2.1326.rv and was introduced into the backbone amplified with the primers GA_pAYCUCAS9.1326.fw and GA_pAYCUCAS9.1326.rv by Gibson assembly. A SapI site in the plasmid backbone was removed in this step. A synthetic construct (Eurofins MWG; see Fig. S2 at https://doi.org/10.6084/m9.figshare.7776818) was designed as described for pCP-tRNA, except that expression of the sgRNA was driven from the A. gossypii *TEF1* promoter and the *CYC1* terminator from S. cerevisiae. The AgeI and SpeI restriction sites flanking the cassette were used for cloning into doubly digested GA_pAYCUCAS9.1326.ARS vector. C. tropicalis guide RNAs were designed using CHOPCHOP v2 (45, 46).

### Transformation of *Candida* strains.

C. parapsilosis CLIB214 and C. metapsilosis SZMC8093 strains were transformed using the lithium acetate method as described in reference 29, with minor modifications. Each repair template was generated by primer extension of overlapping oligonucleotides (see Table S1 at https://doi.org/10.6084/m9.figshare.7776833 and Table S2 at https://doi.org/10.6084/m9.figshare.7776842), and 25 μl of unpurified product was used to transform yeast cells. Transformation of C. tropicalis strains was performed by using a modified electroporation protocol (12, 47, 48). C. tropicalis cells were grown to an *A*_600_ of 5 to 10 and then resuspended in 0.1 M lithium acetate–10 mM Tris-HCl (pH 8.0)–1 mM EDTA–10 mM dithiothreitol (DTT) and incubated at room temperature for 1 h. Cells were washed twice with ice-cold water and once in 1 M ice-cold sorbitol. The sorbitol wash was decanted, and cells were resuspended in the remaining liquid. Approximately 40 to 50 μl of cells was used per transformation, with 5 μg of plasmid together with 5 μg of purified repair template. Cells and DNA were electroporated at 1.8 kV by using a Bio-Rad Pulser XCell Electroporator and immediately resuspended in 1 ml of cold 1 M sorbitol. Cells were subsequently resuspended in 1 ml of YPD and allowed to recover for 4 h at 30°C before plating was performed on selective media (YPD plus 200 μg/ml nourseothricin). Nourseothricin-resistant transformants were patched onto YPD (and SC medium lacking adenine where indicated) and screened by colony PCR. The mutation efficiency was calculated as follows: (edited transformants on the plate) × 100/(total number of transformants on the plate). Representative mutants were sequenced by Sanger sequencing (Eurofins MWG). Loss of the plasmid was induced by patching transformants onto YPD agar without selection and repatching every 48 h until they no longer grew on YPD agar plates containing 200 μg/ml nourseothricin.
