# Correction for Lombardi et al., “Plasmid-Based CRISPR-Cas9 Gene Editing in Multiple *Candida* Species”

**DOI:** 10.1128/mSphere.00494-20

**Published:** 2020-06-10

**Authors:** Lisa Lombardi, João Oliveira-Pacheco, Geraldine Butler

**Affiliations:** a School of Biomolecular and Biomedical Science, Conway Institute, University College Dublin, Belfield, Dublin, Ireland

## AUTHOR CORRECTION

Volume 4, no. 2, e00125-19, 2019, https://doi.org/10.1128/mSphere.00125-19. Figure 4: it has been brought to our attention that there is an error in the plasmid cartoon shown in panel A of this figure. In particular, the promoter that drives expression of CAS9 is *MgACT1*, not *MgTEF1*. The corrected figure is shown below.

**Figure 1:**
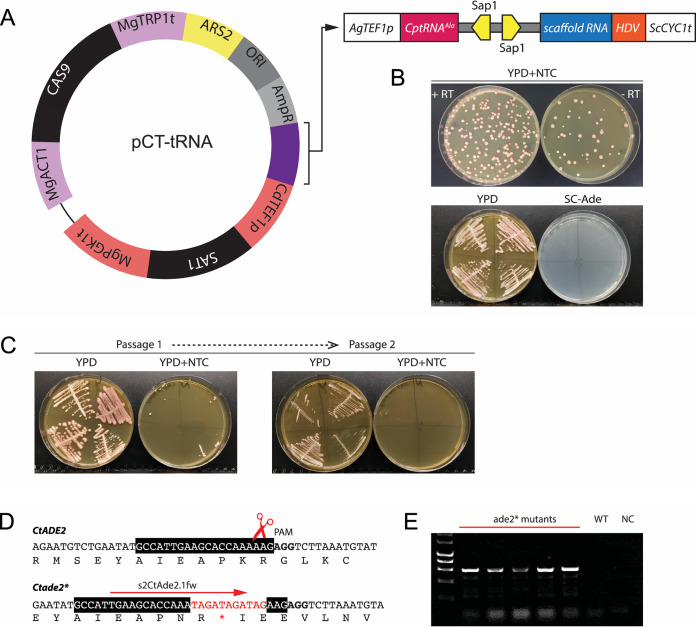


In lines 3 and 4 of the Fig. 4 legend, “the *CAS9* gene is expressed from the *M. guilliermondii TEF1* promoter” should read “the *CAS9* gene is expressed from the *M. guilliermondii ACT1* promoter.”

On page 6 of the PDF, line 9 of the “Designing a plasmid for gene editing in C. tropicalis” paragraph, “placing *CAS9* under the *TEF1* promoter from Meyerozyma guilliermondii” should read “placing *CAS9* under the *ACT1* promoter from Meyerozyma guilliermondii.”

